# “The psychological impact of COVID-19 on medical education of final year students in Pakistan: A cross-sectional study”

**DOI:** 10.1016/j.amsu.2020.11.025

**Published:** 2020-11-12

**Authors:** Adeel Abbas Dhahri, Sohail Yousuf Arain, Ayesha Majeed Memon, Ahsan Rao, Medical Education Pakistan (MEP) collaborator group, Muhammad M. Khan, Gulzam Hafeez, Mehvish A. Dhahri, Faizan G. Mustafa, ShewaRam Malhi, Muhammad H. Iqbal, Raheel Ahmad, Ifra Aziz, Anum S. Arain, Danish Nankani, Muhammad W. Hussain, Muhammad A. Kausar, Muhammad Saqlain, Shilpa Chawla, Hamza Azhar, Hajrah Haneef, Hira S. Arain, Seerat F. Arain, Muhammad S. Shahid, Sania Iqbal, Maryam Mughal, Shariq A. Awan, Hummaz Mehbub, Fahad Qiam, Jazib G. Abro, Talha Khattak, Dujanah S. Bhatti, Abdul M. Choudhary, Abul F.A. Khan, Shehla Baqai, Farooq Afzal, Sharjeel A. Dhahri, Prince A. Dhahri, Samar Ghufran, Izza Umbrin, Waqas Shehdio, Mubashir Shaikh, Maria Dhahri, Mehmood Memon, Yasar Yousufzai, Jai Kumar, Muhammad Ilyas, Tayyab Chaudhary, Sunila Nawaz, Hira Bilal, Naila Faraz, Rajesh Chawla, Sameer Shaikh, Muhammad Amer Mian

**Affiliations:** a The Princess Alexandra Hospital NHS Trust, United Kingdom; b Muhammad Medical College, Pakistan; c Dow University of Medical & Health Sciences, Pakistan; d Cambridge University Hospitals NHS Foundation Trust, UK; e Central Park Medical College, Lahore Pakistan

## Abstract

**Introduction:**

COVID-19 pandemic has resulted in a strong impact on students’ wellbeing, with associated uncertainty about the future. We conducted a cross-sectional survey to assess the psychological effects of COVID-19 on the medical education of final year students in Pakistan.

**Methods:**

We conducted prospective, cross-sectional survey, as a snapshot, from June 07, 2020 till June 16, 2020, among final year medical and dental students. The 20-questions survey questionnaire was based on rating-scale items to focus on psychological symptoms, institutional preparedness for such crisis and confidence in becoming a future doctor. Descriptive statistics were calculated using Multivariate regression analysis.

**Results:**

Majority of participants (n = 1753/2661, 65.9%) were female. Despite timely closure of institutes, delay in the start of the online teaching (beta coefficient 0.08, P-value 0.02) was significantly correlated with the depressive symptoms. A significant percentage of students (n = 1594, 59.9%) wanted a delay in exit exams due to intimidation. A similar proportion of students also lost confidence to be a competent doctor in future which was positively associated with male gender (beta coefficient 0.21, P-value < 0.001).

**Conclusion:**

Our study shows that COVID-19 pandemic has brought significant psychological influence on the medical education of final year students. Despite a stressful crisis, final year medical and dental students are still willing to serve the community. In addition to supporting their emotions and psychological wellbeing, stress counselling, and transforming current medical curricula is crucial to pursue ceaseless medical education and to become a safe future doctor.

## Highlights

• COVID-19 pandemic has brought significant psychological influence on the medical education of final year students.
• A significant proportion of the medical and dental institutions were not well prepared before the lockdown.
• The students' preparation for their exams has been affected by the COVID-19 pandemic.
• Institutes need to establishing online ‘social support group’ with the mental health team to provide supportive therapy.
• Supporting students’ emotions and psychological wellbeing, and transforming current medical curricula is crucial.

## 1 Introduction

Stress is a commonly experienced in response to a threat to adapt to psychological, intellectual or somatic wellbeing [1,2]. It is proven that medical education is characteristically anxiety-ridden and nerve-racking schooling to ease and energize, however, some have suggested a positive impact of stress on learning [3,4]. Most researches relate study stress in medical students to confounding factors like medical institutes, academic obstacles, immense course workload, lack of time off, changes in course curriculum compounded by uncertainty about the state of the exam, lesser preparation time, irregular nutrition habits, sedentary lifestyle and higher parents’ expectations. Situation worsens when there is insufficient and inappropriate practical as well as economic support. While the majority of the medical students fall in the category of moderate stress, female students are found to have an excessive degree of stress. Sequentially, this can result in poor student wellbeing and later compromise in patient safety [[5], [6], [7], [8]].

As COVID-19 pandemic continues to soar emotions in general population, medical students also remain in a state of uncertainty about the future, causing profound psychological effects [7]. During this public health emergency, where COVID-19 effected were filling up hospitals, giving priority to students’ health led to disruption in many regular and routine activities [9]. As WHO implements useful and practical guidelines, like social distancing, many schools and universities suspended their educational activities and proceeded to an online setting. This obviously brought medical students at the point of the prevailing pressure and long-lasting metamorphosis in medical education and upturned the surge in anxiety [7,10,11].

Simultaneously, as uncertainty continues among final year medical students, suspension in clinical exposure may cause harmful effect in their exam and future performance as a doctor [12]. Staying healthier is becoming a new aggravating factor of stress in medical students [9].

We hypothesize that COVID-19 pandemic has adverse effects on students mental-health wellbeing and education. As we assumed that the long-term effects of COVID-19 on the medical students remain a riddle, we conducted this cross-sectional online study intending to assess the psychological effects of COVID-19 pandemic on medical education of the final year medical and dental students in Pakistan.

## 2 Methods

We conducted a nationwide cross-sectional cohort study, as snapshot for 10 days from June 07, 2020, on final year medical and dental students in Pakistan. A 20-question survey form drafted using Google Survey form, for self-completion, was distributed using social media like WhatsApp, Facebook, and emails. All the questions in a survey were mandatory and were drafted by three consultants and three undergraduate medical students, including Professor of Surgery, with interest in Medical education. The questionnaire was developed, on rating-scales items, which focused on demographic details, psychological symptoms related with the closure of their institutes, institutional preparedness for such crisis, confidence in becoming future doctor, and developing symptoms of COVID-19. We enrolled a team of collaborators from throughout the country, volunteering to widely circulate the survey to collect the data from final year students of different, public and private sector, medical and dental colleges. All the final year medical and dental students who were in the middle of their exit exams or awaiting results were excluded from the study.

The study was performed in line with ethical guidelines for internet-mediated research [13]. All the respondents were informed about the objectives of the study including confidentiality of the data. As they consented to volunteer themselves in this completely anonymised, non-experimental, online study, and as no identifiable information was gathered, no ethical approval was required.

The questionnaire is available in the supplementary information.

Sample size was calculated as 376 with 95% confidence level and 5% margin of error. Descriptive statistics were calculated for the survey population. Multivariate regression analysis was carried out to assess the correlation between factors and worse outcomes. The Beta coefficient was used to measure the degree of correlation, and P value of less than 0.05 was considered significant.

This study has been reported in line with the STROCSS criteria [14]. The study has also been retrospectively registered with Research Registry at http://www.researchregistry.com on August 11, 2020 and the unique identifying number is: researchregistry5898 [15].

## 3 Results

Pakistan has 114 registered medical institutes, 44 as public sector while remaining 70 as the private sector. Fifty-five institutes are providing dental education in Pakistan, out of these 15 are public sector while 40 are running as private sector institutes. In total, there are about 15900 students in these medical and dental institutes; 13600 in medical colleges or universities while 2300 in dental institutes [16].

**Fig. 1:**
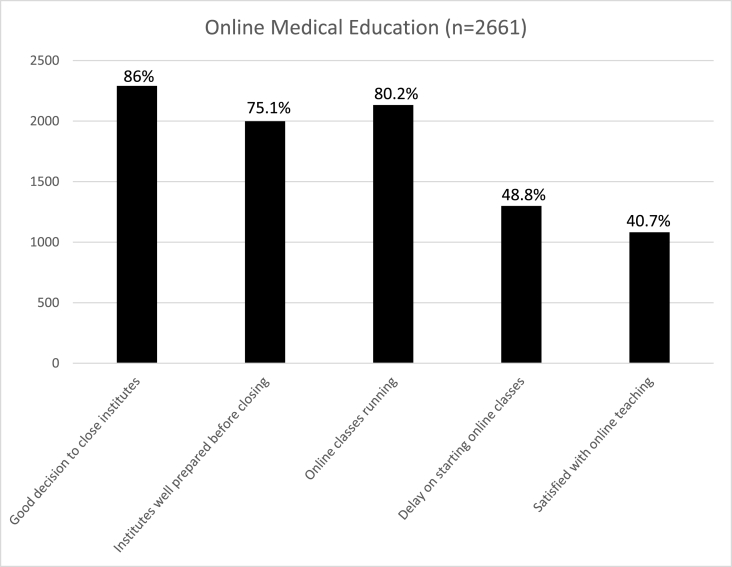
Online medical education.

There were a total of 2661 participants in this qualitative study, who responded to the online questionnaire. There were 1753 (65.9%) female while 908 (34.1%) male participants and most of the participants belonged to government-based medical institute (n = 1624, 61.0%). Most of the participants were from Punjab province (n = 1494, 56.1%), followed by Sindh (n = 798, 29.9%), Khyber Pakhtunkhwa (n = 287, 10.7%), Baluchistan (n = 46, 1.7%), and Azad Kashmir (n = 36, 1.4%). Most of the students were studying undergraduate medicine (n = 2264, 85.1%), and the rest were studying undergraduate dentistry (n = 398, 14.9%) (Table 1). Most of the educational institutions (n = 2486, 93.4%) closed timely and appropriately during COVID-19 pandemic. A large proportion of the students (n = 292, 11.1%) also displayed symptoms of COVID-19. Most of the students (n = 2291, 86%) agree that closing down the institution was correct during COVID-19 pandemic; however, a significant proportion of them (n = 662, 24.9%) felt that the institutions were not well prepared before the lockdown (Fig. 1).

**Table 1:** Demographics of participants.

	Male	Female
*1. **Participants***	908 (34.1%)	1753 (65.9%)
*2. **Study group***		
a. Medicine (MBBS) (n = 2263, 85.1%)	782	1481
b. Dentistry (BDS) (n = 398, 14.9%)	126	272
*3. **Sector***		
a. Public (n = 1623, 61%)	506	1117
b. Private (n = 1038, %)	402	636
*4. **Location of institute***		
a. Punjab (n = 1494, 56.1%)	500	994
b. Sindh (n = 798, 29.9%)	205	593
c. Khyber Pakhtunkhwa (n = 287, 10.7%)	158	129
d. Baluchistan (n = 46, 1.7%)	30	16
e. Azad Kashmir (n = 36, 1.4%)	15	21

**Fig. 2:**
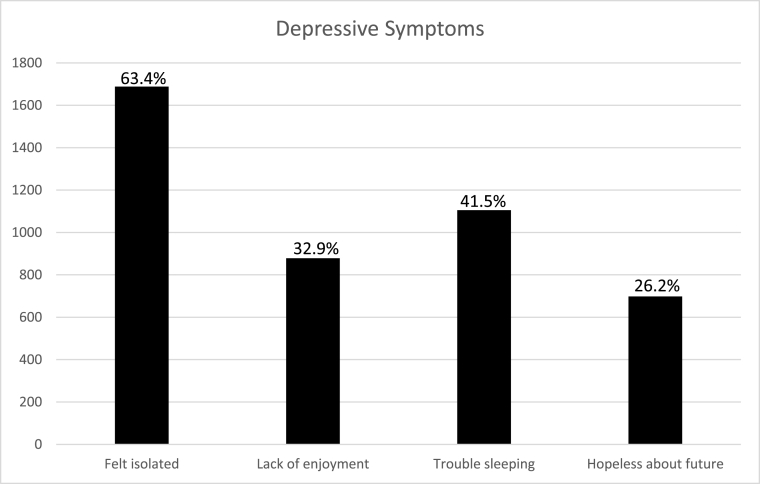
Depressive symptoms.

The large proportion of the students displayed depressive symptoms. They felt isolated (n = 1688, 63.4%), experienced lack of enjoyment in daily activities (n = 878, 32.9%), had problems with sleeping very frequently (n = 1105, 41.5%), and were not hopeful about their future (n = 698, 26.2%) (Fig. 2). Following multivariate analysis, having COVID-19 symptoms (beta coefficient 0.11, P-value 0.05), studying in a private institution (beta coefficient 0.1, P-value 0.01) and delay in the start of the online training by the institution (beta coefficient 0.08, P-value 0.02) were the factors that were significantly and positively correlated with the depressive symptoms. Gender (P-value 0.2), studying in a smaller province (P-value 0.9), and studying in a dental program (P-value 0.79) did not have any significant correlation with the depressive symptoms.

Majority of the educational institutions (n = 2133, 80.1%) started online courses and education, however, a large proportion of the students (n = 1299, 48.8%) thought that there was a delay by the institutions to start online classes and they (n = 1579, 59.3%) were not satisfied with the set-up of the online teaching (Fig. 1).

**Fig. 3:**
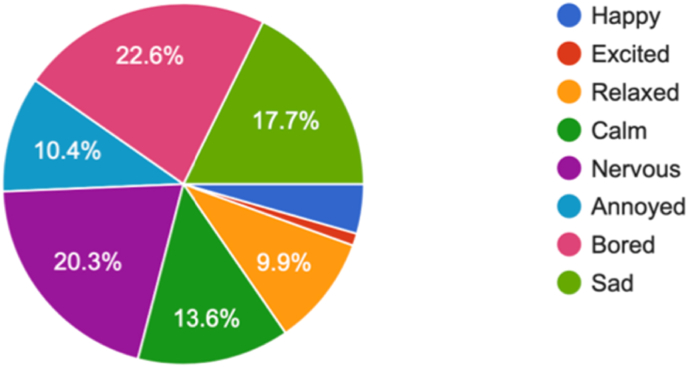
What are your feelings on the closure of your institute?.

A fair proportion of the students felt bored or nervous on the closure of their institute (n = 601, 22.6% vs n = 540, 20.3% respectively). Sadness (n = 471, 17.7%) was also evident while some were annoyed (n = 276, 10.4%) with this decision of closure of the institute. Rest of the students either felt relaxed, clam or happy with such a decision (Fig. 3).

The students' preparation for their exams was also affected by the COVID-19 pandemic. Although they did not have on-site conventional teaching during COVID-19, they still did not find time for self-study (n = 1991, 74.8%) and did not feel confident to sit in the final exams (n = 2364, 88.8%) and (n = 1594, 59.9%) wanted a delay for the exam date. The large proportion of students (n = 1463, 54.9%) thought the pandemic would deteriorate their ability and confidence to be a competent doctor in the near future (Table 2). The feeling of lack of confidence to be a competent doctor was positively associated with male gender (beta coefficient 0.21, P-value < 0.001), and studying in a smaller province (beta coefficient 0.08, P-value 0.01). It had a significantly negative correlation with studying in a dental program (beta coefficient −0.06, P-value 0.02) and having COVID-19 symptoms (beta coefficient −0.06, P-value 0.03).

**Table 2:** Students’ self-reflection on their future.

	Yes	No
***Finding time for self-study***	670, 25.2%	1991, 74.8%
***Confident to sit exit exam***	297, 11.2%	2364, 88.8%
***Pattern of exam***		
a. Same exam pattern as pre-COVID-19	1672, 62.8%	–
b. Online only	378, 14.2%	–
c. Combined face-to-face & online pattern	611, 22.9%	–
***Delay in exit exam***	1594, 59.9%	1067, 40.1%
***Confident to work as doctor***	1198, 45.1%	1463, 54.9%

When asked about the preference about the type of final exit exam, a majority of the students (n = 1672, 62.8%) preferred to have same exam pattern as before during pre-COVID-19 era. Few (n = 378, 14.2%) showed interest in online examination pattern while slightly higher number (n = 611, 22.9%) were interested in both online with face-to-face clinical and objective structured clinical examination (OSCE) exams (Table 2).

## 4 Discussion

Our study focused on the final year students of both medical and dental institutes in Pakistan during times of COVID-19 pandemic.

### 4.1 Closure of institutes

As COVID-19 pandemic forced rapid transformation within societies, the educational system also faced challenges, noticeably medical institutions, throughout the world. In response to COVID-19 crisis, like the rest of the world, medical institutes in Pakistan also started to close down from the mid of March 2020 with little variation, aimed social distancing. This was the reflection of successful responses witnessed in the past to alleviate disease spread effectively [[17], [18], [19]]. In our survey, although a minority of students claim that their institute did not close timely during COVID-19 crisis, most of these students were from private sector institutes.

There is no data available to claim the effect of institutional closure on the control of transmission of COVID-19 and mental health outcomes. Department of Health and Social Care UK has launched a study in order to find the incidence of COVID-19 in schools [18,20]. We found that a good number of students displayed the symptoms of COVID-19 infection, but there was no significant correlation between delayed institute closure and COVID-19 prevalence. However, the majority of the students in our survey agreed that it was a good decision to close down their institute because of COVID-19 pandemic. A need to understand medical institutions closure on tomorrow's doctor is the utmost need of the time to avoid long-term iceberg effects.

### 4.2 Psychological impact

Most literature has proven that depressive symptoms are more common in medical students as compared to the general population, even more, common in the female gender. This can be related to a biological difference in females as compared to male [[21], [22], [23], [24], [25]]. Our data did show any significant difference in both genders. Medical or Dental career for undergraduate students is very challenging and stressful, both physically and mentally. Being selected in highly demanding career does not mean that students are resilient to stress-related conditions. As during any stress-related situation, COVID-19 has already brought significant stress crisis in the population [26,27]. Our study also identified such stress-related symptoms in undergraduates, including nervousness and sadness. Such level of stress can bring a significant negative impact on resilience and strength of our future doctors to cope with crisis situations like a difficult patient and complicated surgery. Resilience training has shown more extensive benefits to improve performance during different interventions and can be incorporated in the undergraduate curriculum of medical and dental students [28].

Depressive symptoms were commonly seen in those students who witnessed late start of their online classes. We also identified that depressive symptoms were more evident in students studying at the private institutes. It was also found that most of these students had COVID-19 symptoms as well. As mentioned in literature, about correlation between COVID-19 and psychological symptoms, this may be the answer to our results [29].

During the survey, when asked about feeling related with the closure of their institutes, the answers were comparable with eight basic mood types described by Desmet et al., in 2012 and Watson and Tellegen 1985 [30,31]. Most of our students felt bored or nervous on the closure of their institute while others felt sad or annoyed. However, few of the students welcomed the decision of closure of institutes in a positive way when they felt relaxed, calm or happy. The corresponding author described such different moods as ‘wheel of self-colour’ which seems to be blending during times of crisis like COVID-19 pandemic (Fig. 3).

### 4.3 Future doctors

The final year during undergraduate studies is very demanding and more patient-centred. During COVID-19 related lock-down, at the times of uncertainty, closure of medical institutions cancelled all clinical activities tangled by lack of preparedness of some institutions [12]. This brought many psychological impacts, including failure to focus on self-study and preparedness for final year exit exams to become tomorrow's safe and competent doctors, leading to deterioration in self-confidence and self-dependence. This may lead to the fiasco in being ready to work at any place outside their parent country. Although stress related symptoms being more common in the female gender, our study also found that such a feeling of lack of confidence was more evident in male students. A recent survey conducted in 32 UK medical schools found that COVID-19 has significantly impacted confidence and willingness to work in a hospital. This can generally affect smooth change from student to doctor life [32].

### 4.4 Burn-out

Long-term psychological effects of the COVID-19 are yet to be understood and explained at a massive scale. Burn-out is another crisis another iceberg waiting to hit the student population. This can be related to expected prolonged stress-related with many factors described above. If happens, it can lead to both physical and mental draining. To understand this significant health hazard and to enhance stress coping abilities, stakeholders in Pakistan should take responsibilities of the wellbeing of their students. Changes can be brought by reforming and bringing positive improvements to the current curriculum with practices to improve personal measures like better communications and interactions at all levels, counselling and advisory services to widen their confidence in becoming a safe future doctor. Such measures to cope with stress during undergraduate schooling has been suggested in literature [25,[33], [34], [35]].

To the best of our knowledge, this is the first study examining the psychological perspective of COVID-19 on final year medical and dental students. As this survey was to evaluate such psychological impact, we focused on final year students because their curriculum has clinical training as an integral part and as they are the most vulnerable population, having a professional career ahead to start. It will be more beneficial to conduct further follow-up survey after the pandemic to assess the long-term consequences of it on them.

Crisis in any form is related to different forms of mood, especially stress and anxiety. As COVID-19 has already transformed our lives, there is an absolute need for long-term plan to prepare for the crisis now and for the future to avoid negative impact on the education with less psychological stress-related effects on the medical students. Institutes should adopt time-management strategies and take the responsibilities of their students, to start a program which can be virtual during the immediate current pandemic situation. As students are more active in using social media, they should take advantage of this opportunity to establish online ‘social support group’ to establish either as a group or one-to-one support therapy with the mental health team, which may further suggest cognitive behaviour therapy. Currently, some institutes have initiated such supportive measures, but its status is yet to be confirmed on a large scale.

We also suggest reviewing the undergraduate curriculum with a reflection on stress counselling and management of the students in periods of sudden transitions. Resilience training may be an integral part of their curriculum. It is also suggested that the government and medical institutions should collaborate to resolve this problem, by launching visionary document as guidance, for example, in order to provide high-quality, timely crisis-oriented psychological services to college students.

## 5 Conclusion

COVID-19 pandemic keeps transforming our life and health, many lessons are as yet to be understood. What will be the long-term effect is still a mystery, but it has brought significant physical and psychological changes in the life of a future doctor. Although in the middle of nowhere and during the peak times of stressful crisis, final year medical and dental students are still willing to serve the community. Supporting their emotions and wellbeing, by establishing a social support group, for example, is vital and crucial as they prepare for their exit exams. While the medical or dental student is expected to be resilient and irrepressible during stress filled situation, current curricula do not stress such scenarios. Proper grooming and training during the undergraduate period can result in a safe and secure future doctor with better patient outcomes in the future. During the current scenario, reviewing our policies is the utmost priority.

### Annals of medicine and surgery

The following information is required for submission. Please note that failure to respond to these questions/statements will mean your submission will be returned. If you have nothing to declare in any of these categories then this should be stated.

Please state any conflicts of interest.

All authors must disclose any financial and personal relationships with other people or organizations that could inappropriately influence (bias) their work. Examples of potential conflicts of interest include employment, consultancies, stock ownership, honoraria, paid expert testimony, patent applications/registrations, and grants or other funding.

There was no conflict of interest or involvement in any organization.

Please state any sources of funding for your research.

All sources of funding should be declared as an acknowledgement at the end of the text. Authors should declare the role of study sponsors, if any, in the collection, analysis and interpretation of data; in the writing of the manuscript; and in the decision to submit the manuscript for publication. If the study sponsors had no such involvement, the authors should so state.

This study did not receive any grant from any funding agencies in public or organizations.

## Ethical Approval

Research studies involving patients require ethical approval. Please state whether approval has been given, name the relevant ethics committee and the state the reference number for their judgement.

The study was performed in line with ethical guidelines for internet-mediated research.

All the respondents (final year students) were informed about the objectives of the study including confidentiality of the data. As they consented to volunteer themselves in this completely anonymised, non-experimental, online study, and as no identifiable information was gathered, no ethical approval was required.

## Consent

Studies on patients or volunteers require ethics committee approval and fully informed written consent which should be documented in the paper.

Authors must obtain written and signed consent to publish a case report from the patient (or, where applicable, the patient's guardian or next of kin) prior to submission. We ask Authors to confirm as part of the submission process that such consent has been obtained, and the manuscript must include a statement to this effect in a consent section at the end of the manuscript, as follows: “Written informed consent was obtained from the patient for publication of this case report and accompanying images. A copy of the written consent is available for review by the Editor-in-Chief of this journal on request”.

Patients have a right to privacy. Patients’ and volunteers' names, initials, or hospital numbers should not be used. Images of patients or volunteers should not be used unless the information is essential for scientific purposes and explicit permission has been given as part of the consent. If such consent is made subject to any conditions, the Editor in Chief must be made aware of all such conditions.

Even where consent has been given, identifying details should be omitted if they are not essential. If identifying characteristics are altered to protect anonymity, such as in genetic pedigrees, authors should provide assurance that alterations do not distort scientific meaning and editors should so note.

## Author contribution

Please specify the contribution of each author to the paper, e.g. study concept or design, data collection, data analysis or interpretation, writing the paper, others, who have contributed in other ways should be listed as contributors.

AAD and SYA: Study design, questionnaire drafting: analysis and writing, AMM: Questionnaire drafting: data collection and writing, AR: Formal analysis, writing and editing

Medical Education Pakistan (MEP) collaborator group (MMK et al., Medical Education Pakistan (MEP) collaborator group (MMK, GH, MAD, FGM, SRM, MHI, RA, IA, ASA, DN, MWH, MAK, MS, SC, HA, HH, HAS, SFA, MSS, SI, MM, SAA, HM, FQ, JGA, TK, DSB, AMC, AFAK, SB, FA, SAD, PAD, SG, IU, WS, MS, MD, MM, YY, JK, MI, TC, SN, HB, NF, RC, SS): Data collection.

MAM: Supervision, Questionnaire drafting, Data collection, Editing

## Registration of research studies

In accordance with the Declaration of Helsinki 2013, all research involving human participants has to be registered in a publicly accessible database. Please enter the name of the registry and the unique identifying number (UIN) of your study.

You can register any type of research at http://www.researchregistry.com to obtain your UIN if you have not already registered. This is mandatory for human studies only. Trials and certain observational research can also be registered elsewhere such as: ClinicalTrials.gov or ISRCTN or numerous other registries.

## Name of the registry

Research Registry.

## Unique Identifying number or registration ID

Researchregistry5898.

3. Hyperlink to your specific registration (must be publicly accessible and will be checked): https://www.researchregistry.com/browse-the-registry#home/registrationdetails/5f3316f4d5c27100153034a7/

## Guarantor

The Guarantor is the one or more people who accept full responsibility for the work and/or the conduct of the study, had access to the data, and controlled the decision to publish.

## Author agreement

This is to confirm that the manuscript title “The psychological impact of COVID-19 on medical education of final year students in Pakistan: a cross-sectional study” has been read and approved by all authors. During writing, all the authorship requirements were met and believe that the manuscript is an honest work.

## Ethical approval, Consent and data availability

All the respondents (final year students) were informed about the objectives of the study including confidentiality of the data. As they volunteered themselves in this non-experimental study, no ethical approval was required.

## Consent for publication

All the contributing authors and participants have consented for publication.

## Funding

This study did not receive any grant from any funding agencies in public or organizations.

## Provenance and peer review

Not commissioned, externally peer reviewed.

## Declaration ofcompeting interest

There was no conflict of interest or involvement in any organization.
